# A nomogram based on trauma-induced coagulopathy for predicting hospital mortality in multi-trauma patients: a retrospective study

**DOI:** 10.1007/s11739-025-03867-w

**Published:** 2025-01-28

**Authors:** Shaochuan Chen, Jiale Yang, Xuezhi Shi, Anwei Liu, Guodong Lin, Huasheng Tong

**Affiliations:** 1 Department of Emergency Medicine, Huiyang Sanhe Hospital, Huizhou, China; 2 Guangzhou University of Chinese Medicine, Guangzhou, China; 3 Department of Intensive Care Unit, General Hospital of Southern Theatre Command of PLA, Guangzhou, China

**Keywords:** Multi-trauma, Trauma-induced coagulopathy, Prognosis, Nomogram

## Abstract

Multi-trauma presents significant challenges due to the complexity of injuries and high mortality rates. Early identification and intervention are crucial for improving outcomes in these critically injured patients. This retrospective study analyzed clinical data from multi-trauma patients admitted to the emergency department of Huiyang Sanhe Hospital between January 10, 2020, and September 30, 2022. Univariate and multivariate logistic regression analyses were conducted to identify independent predictors of hospital mortality. A prediction model was developed based on these prognostic markers, visualized using a nomogram, and its discriminative ability and clinical benefit were evaluated. A total of 124 multi-trauma patients were included in the study, with a hospital mortality rate of 26.7%. Univariate and multivariate logistic regression analyses identified trauma-induced coagulopathy (TIC) (OR 4.238, 95% CI 1.46–12.28), blood urea nitrogen (BUN) (OR 1.397, 95% CI 1.09–1.78), and Glasgow Coma Scale (GCS) score (OR 0.720, 95% CI 0.61–0.85) as independent factors of hospital mortality. Therefore, a nomogram incorporating TIC, BUN, and GCS score was constructed and demonstrated excellent predictive performance and clinical impact (AUC 0.898, 95% CI 0.834–0.962). The nomogram developed in this study provided a practical tool for early prediction of hospital mortality in multi-trauma patients. By focusing on TIC, BUN, and GCS score, this model may facilitate rapid bedside assessment and timely intervention. However, further multicenter, prospective studies are required to validate its performance and applicability.

**Supplementary Information:**

The online version contains supplementary material available at 10.1007/s11739-025-03867-w.

## Introduction

Trauma remains a major public health challenge globally, affecting individuals across all age groups [1]. With the rapid pace of economic development, urbanization, and lifestyle changes, the incidence and mortality rates of trauma have been increasing. Trauma accounts for approximately 7% of all deaths in China from 2000 to 2017, making it the fifth leading cause of death, following malignant tumors, cerebrovascular diseases, respiratory diseases, and heart conditions [2], underscoring the critical need for early intervention and effective management in trauma patients.

Severe injuries often result in multi-trauma, where multiple anatomical sites are simultaneously wounded due to high-energy mechanical forces. The complexity of these injuries and their associated high mortality rates necessitate precise assessment and timely treatment [3]. Traditionally, the Injury Severity Score (ISS) has been used to evaluate the severity of injuries and predict mortality risk in trauma patients [4]. However, the ISS has some limitations, such as being influenced by the subjective judgment of physicians and focusing only on the physical injuries in specific body regions [5], which makes it less effective in predicting the prognoses of multi-trauma patients during the early stages of resuscitation.

Moreover, multi-trauma frequently leads to metabolic dysfunction and severe physiological disturbances, which are not adequately captured by the ISS [6]. Recognizing these limitations, researchers have proposed a more comprehensive definition of multi-trauma that combines anatomical injuries with physiological factors [7]. This updated definition includes key indicators such as coagulation disorders, hypotension, altered consciousness, acidosis, and advanced age. Among these factors, trauma-induced coagulopathy (TIC) particularly plays a crucial role in the rapid progression of injuries and contribute to high clinical mortality rates [8].

Early identification and intervention during the “golden first hour” are vital for reducing trauma-related complications and mortality [9]. After multi-trauma, the body undergoes significant metabolic and stress responses, leading to a cascade of events including hormonal imbalances, systemic inflammatory responses, and coagulation activation [10]. These processes can result in cellular damage, hypoxia, and complications like acidosis and hypothermia, creating a vicious cycle that worsens patient outcomes [10]. Therefore, rapid and effective management of multi-trauma patients after injury is essential for maintaining a stable internal environment, reducing the metabolic response, and decreasing clinical mortality.

Recent studies have identified several independent risk factors for mortality in multi-trauma patients, such as lactate levels, coagulation abnormalities, and the Glasgow Coma Scale (GCS) score [11–13]. However, few clinical research focused on constructing a prediction model by combing critical independent factors for prognosis evaluation in multi-trauma patients. Given the need for rapid and accurate assessments in emergency settings, this study aims to identify independent risk factors and develop a prediction model for hospital mortality by evaluating factors such as TIC, injury severity, and physiological responses, thus ultimately improving early emergency management of multi-trauma patients.

## Methods

### Patients

A total of 150 patients with multiple injuries admitted to the emergency department of Huiyang Sanhe Hospital from January 1, 2020, to September 30, 2022, were retrospectively enrolled. The diagnostic criteria for multi-trauma were as follows: (1) patients who met the clinical diagnostic criteria for trauma and had an ISS score of ≥ 16; and (2) patients with injuries in ≥ 2 locations [14]. All patients were provided with comprehensive monitoring, care, and treatment. The Ethics Committee of Huiyang Sanhe Hospital approved this retrospective study and waived the requirement for informed consent.

### Inclusion and exclusion criteria

**Fig. 1:**
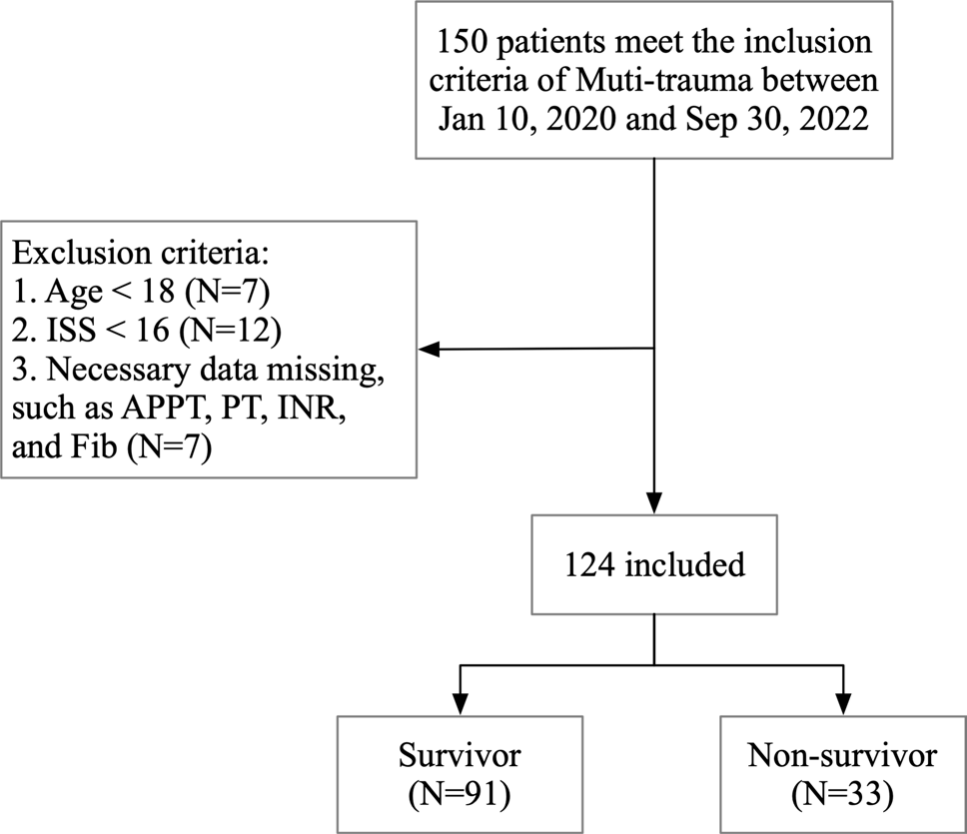
Flow chart illustrating inclusion and exclusion of patients with multi-trauma

The inclusion criterion was that patients met the diagnostic criteria of multi-trauma. The exclusion criteria were as follows: (1) patients who were not enrolled within 6 h after trauma onset; (2) age < 18 years; (3) patients in the pregnancy or lactation period; (4) patients with a history of hematologic disorders or tumor diseases; (5) recent use of anticoagulation and antiplatelet drugs; and (6) missing data. The clinical data of 150 patients who met the diagnostic criteria for multi-trauma were recorded, and 124 patients were included in the study, consisting of 91 survivors and 33 non-survivors (Fig. 1).

### Clinical and laboratory data

Data from the electronic medical record system were collected at admission, including (1) demographics: age, gender, and mechanism of injury; (2) vital signs: temperature, heart rate, mean arterial pressure (MAP); (3) routine blood test: white blood cell count (WBC), neutrophil count (Neu), hemoglobin (HB), platelet count (PLT); (4) coagulation function indexes: activated partial thromboplastin time (APTT), prothrombin time (PT), international normalized ratio (INR), and fibrinogen (Fib) levels; (5) other laboratory factors: serum creatinine (Scr), blood urea nitrogen (BUN), and lactate (Lac) levels; (5) severity scores: Injury Severity Score (ISS), Glasgow Coma Scale (GCS) score, Acute Physiology and Chronic Health Evaluation II (APACHE II) score, Sequential Organ Failure Assessment (SOFA) score; (6) interventions: use of vasoactive drugs, mechanical ventilation, continuous renal replacement therapy (CRRT), and transfusion of red blood cell, plasma, platelet, or cryoprecipitate; and (7) others: shock index, traumatic shock, trauma-induced coagulopathy (TIC). TIC was diagnosed based on following criteria: a potential bleeding tendency, requiring supplement of blood products, 50% prolongation of APTT or PT, an INR ≥ 1.5, or Fib < 1g/L [15].

### Statistical analysis

Continuous variables were presented as either mean ± standard deviation for normally distributed data or as median with interquartile range (IQR) for skewed data. Comparisons between groups were conducted using Student’s t-test for normally distributed variables or the Mann–Whitney U test for skewed variables. Categorical variables were expressed as counts and percentages, with comparisons made using the Chi-square test or Fisher’s exact test, depending on the context. To identify factors influencing hospital mortality among multi-trauma patients and develop a prediction model, univariate and multivariate logistic regression analyses were performed. Receiver operating characteristic (ROC) curve analysis was performed for each indicator, calculate the area under the curve (AUC), determine cutoff values, and calculate the sensitivity, specificity, and Youden index. DeLong’s test was used to compare the AUCs of different ROC curves. The “rms” package in R (version 4.2.2) was utilized to plot the calibration curve and create the nomogram, while decision curve analysis (DCA) and the clinical impact curve (CIC) were conducted using the “rmda” package. A two-tailed P-value < 0.05 was considered statistically significant.

## Results

### Baseline characteristics of patients with multi-trauma

A total of 124 patients with median age of 45.00 were enrolled. Among them, 29 (23.40%) was female, and 95 (76.60%) were male. The injury mechanisms included car accidents (76/61.30%), fall from heights (31/25.00%), and other causes (17/13.70%), respectively. The hospital mortality was 26.7% (33/124). Compared with survivors, non-survivors demonstrated higher levels of APTT, PT, INR, Scr, and BUN, and lower levels of admission temperature, MAP, Fib, and PLT, which indicated a more severe coagulopathy combined with unstable vital signs. A greater incidence of TIC (63.60% vs. 19.80%, P < 0.001) and platelet transfusion (33.30% vs. 8.80%, P = 0.001) were found in non-survivors than survivors, with higher chance of receiving vasoactive drug and CRRT treatment. Furthermore, non-survivors exhibited significantly higher ISS, APACHE II, and SOFA scores with lower GCS score (all P-value < 0.001) (Table 1).

**Table 1:** Baseline characteristics of patients with multi-trauma in survivor and non-survivor groups

	Total cases (n = 124)	Survivors (n = 91)	Non-survivors (n = 33)	P-value
Age	45.00 [30.75, 55.00]	41.00 [30.00, 53.00]	55.00 [43.00, 63.00]	0.003
Gender				1.000
Female (%)	29 (23.40%)	21 (23.10%)	8 (24.20%)	
Male (%)	95 (76.60%)	70 (76.90%)	25(75.80%)	
Injury mechanism				0.572
Car accident (%)	76 (61.30%)	54 (59.30%)	22 (66.70%)	
Fall from height (%)	31 (25.00%)	25(27.50%)	6 (18.20%)	
Others (%)	17 (13.70%)	12 (13.20%)	5 (15.20%)	
Temperature				< 0.001
≤ 35℃	40 (32.3%)	16 (17.6%)	24 (72.7%)	
35.1–36.2℃	40 (32.3%)	34 (37.4%)	6 (18.2%)	
≥ 36.3℃	44 (35.5%)	41 (45.1%)	3 (9.1%)	
Heart rate (BMP)	102.76 ± 25.54	103.81 ± 23.40	99.85 ± 30.92	0.447
MAP (mmHg)	76.35 ± 24.82	80.90 ± 23.95	63.80 ± 23.10	0.001
WBC (× 10^9^/L)	17.16 ± 7.14	17.75 ± 6.94	15.53 ± 7.54	0.128
Neu (× 10^9^/L)	13.83 ± 6.49	14.38 ± 6.30	12.31 ± 6.88	0.118
Lac (mmol/L)	4.90 [2.65, 7.08]	4.20 [2.25, 6.25]	5.10 [3.20, 9.90]	0.043
HB (g/L)	116.20 ± 28.17	119.22 ± 27.34	107.88 ± 29.18	0.047
PLT (× 10^9^/L)	172.50 [108.25, 235.75]	185.00 [127.00, 245.50]	121.00 [66.00, 184.00]	0.001
APTT (s)	28.10 [23.67, 35.78]	26.00 [22.90, 33.70]	32.50 [28.20, 46.50]	< 0.001
PT (s)	13.25 [11.80, 16.12]	12.70 [11.55, 14.85]	16.90 [13.90, 18.80]	< 0.001
INR	1.60 [1.01, 2.26]	1.71 [1.23, 2.34]	0.97 [0.67, 1.69]	< 0.001
Fib (g/L)	86.45 [68.95, 116.55]	81.30 [68.20, 108.75]	110.00 [73.70, 148.10]	0.006
Scr (μmol/L)	5.25 [4.10, 6.51]	4.78 [3.95, 6.18]	5.98 [5.10, 7.50]	0.001
BUN (mmol/L)	0.96 [0.73, 1.35]	0.90 [0.70, 1.29]	1.14 [0.83, 1.60]	0.054
Shock index	4.90 [2.65, 7.08]	4.20 [2.25, 6.25]	5.10 [3.20, 9.90]	0.043
Traumatic Shock (%)	60 (48.40%)	40 (44.40%)	20 (60.60%)	0.151
TIC (%)	39 (31.5%)	18 (19.8%)	21 (63.6%)	< 0.001
Mechanical ventilation (%)	72 (58.1%)	41 (45.1%)	31 (93.9%)	< 0.001
Vasoactive drugs (%)	59 (47.60%)	31 (34.10%)	28 (84.80%)	< 0.001
CRRT (%)	16 (12.90%)	4(4.40%)	12 (36.40%)	< 0.001
Red blood cell transfusion (%)	86(69.40%)	60(65.90%)	26(78.80%)	0.249
Plasma infusion (%)	65(52.40%)	43(47.30%)	22(66.70%)	0.087
Platelet transfusion (%)	19 (15.30%)	8 (8.80%)	11 (33.30%)	0.002
Cryoprecipitate transfusion (%)	28 (22.60%)	20 (22.00%)	8 (24.20%)	0.981
ISS score	33.00 [25.00, 41.00]	32.00 [25.00, 37.00]	38.00 [33.00, 48.00]	< 0.001
GCS score	7.00 [3.00, 14.00]	12.00 [5.00, 15.00]	3.00 [3.00, 4.00]	< 0.001
APACHE II score	20.00 [15.00, 30.00]	17.00 [14.00, 22.50]	34.00 [29.00, 37.00]	< 0.001
SOFA score	6.50 [3.00, 9.00]	5.00 [2.00, 8.00]	10.00 [9.00, 14.00]	< 0.001
Degree of Coma				< 0.001
Light (%)	44 (35.5%)	43 (47.3%)	1 (3.0%)	
Moderate (%)	14 (11.3%)	12 (13.2%)	2 (6.1%)	
Severe (%)	66 (53.2%)	36 (39.6%)	30 (90.9%)	
Degree of injury				0.016
Moderate (%)	23 (18.5%)	22 (24.2%)	1 (3.0%)	
Severe (%)	101 (81.5%)	69 (75.8%)	32 (97.0%)	

Data are expressed as median (interquartile range), number (percentage) or mean with standard deviation

Degree of Coma: light GCS 13–15, moderate GSC 9–12, severe GCS ≤ 8; degree of injury: moderate ISS 16–24, severe ISS ≥ 25

*MAP* mean artery pressure, *WBC* white blood cell count, *Neu* neutrophil count, *Lac* lactate, *HB* hemoglobin, *PLT* platelet, *APTT* activated partial thromboplastin time, *PT* prothrombin time, *INR* international normalized ratio, *Fib* fibrinogen, *Scr* serum creatinine, *BUN* blood urea nitrogen, *TIC* trauma induced coagulopathy, *CRRT* continuous renal replacement therapy, *ISS* injury severity score, *GCS* Glasgow Coma Scale, *APACHE* *II* Acute Physiology and Chronic Health Evaluation II, *SOFA* Sequential Organ Failure Assessment

### TIC independently predict hospital mortality

Univariate logistic regression analyses revealed correlations between hospital mortality and risk factors (temperature ≤ 35℃, Lac, APTT, PT, INR, Scr, BUN, TIC, mechanical ventilation, vasoactive drugs, CRRT, platelet transfusion, ISS, APACHE II score, SOFA scores, severe coma, and severe injury) and protective factors (MAP, PLT, Fib, and GCS) (Table S1). After multivariate logistic regression (Forward LR), TIC (OR 4.238, 95% CI 1.46–12.28, P = 0.008) and BUN (OR 1.397, 95% CI 1.09–1.78, P = 0.008) were identified as independent risk factors, while GCS score (OR 0.720, 95% CI 0.61–0.85, P < 0.001) was identified as independent protective factor (Table S2). Accordingly, we constructed a prediction model based on TIC, BUN and GCS score (Table S2). The equation is as follows:

$$\left[logit (\text{ODDs})= -1.444+1.444\times \text{TIC}-0.329\times \text{GCS}+0.334\times \text{BUN}\right]$$

$$\left[\text{Hospital mortality}=\frac{\text{ODDs}}{1+\text{ODDs}}\right]$$

### The performance of the prediction model for evaluating prognoses

**Fig. 2:**
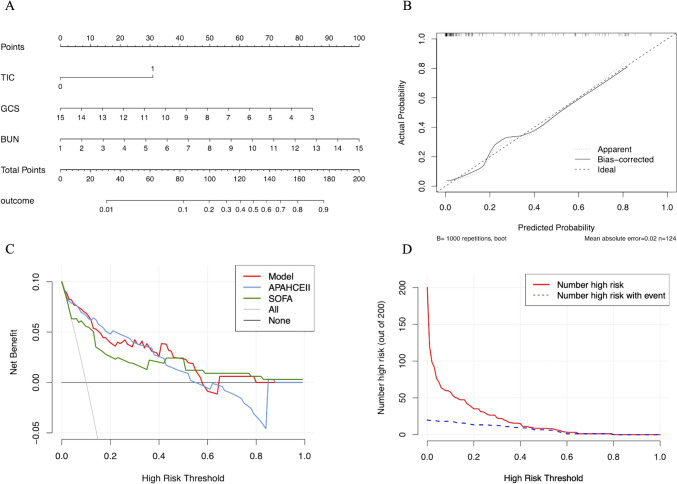
Comprehensive evaluation of the prediction model. **A** nomogram of the prediction model; **B** calibration curve of prediction model; **C** decision curve analysis (DCA) comparison of the prediction model with APACHE II and SOFA scores; **D** clinical impact curve (CIC) of the prediction model

A nomogram and calibration curve were constructed based on the prediction model (Fig. 2A, B). The calibration curve demonstrated satisfactory agreement between the predicted and actual probabilities. The Hosmer–Lemeshow test confirmed that the prediction model was well-fitted (χ^2^ = 9.8576, df = 8, P = 0.2752 > 0.05). As shown in DCA (Fig. 2C), the prediction model provided a greater clinical net benefit compared to both the “treat-all” and “treat-none” strategies across a wide range of threshold probabilities. Furthermore, the CIC (Fig. 2D) revealed a robust consistency between the high-risk patients identified by the model and those who experienced adverse outcomes. Overall, the prediction model demonstrated superior clinical net benefit and satisfactory clinical impact.

**Fig. 3:**
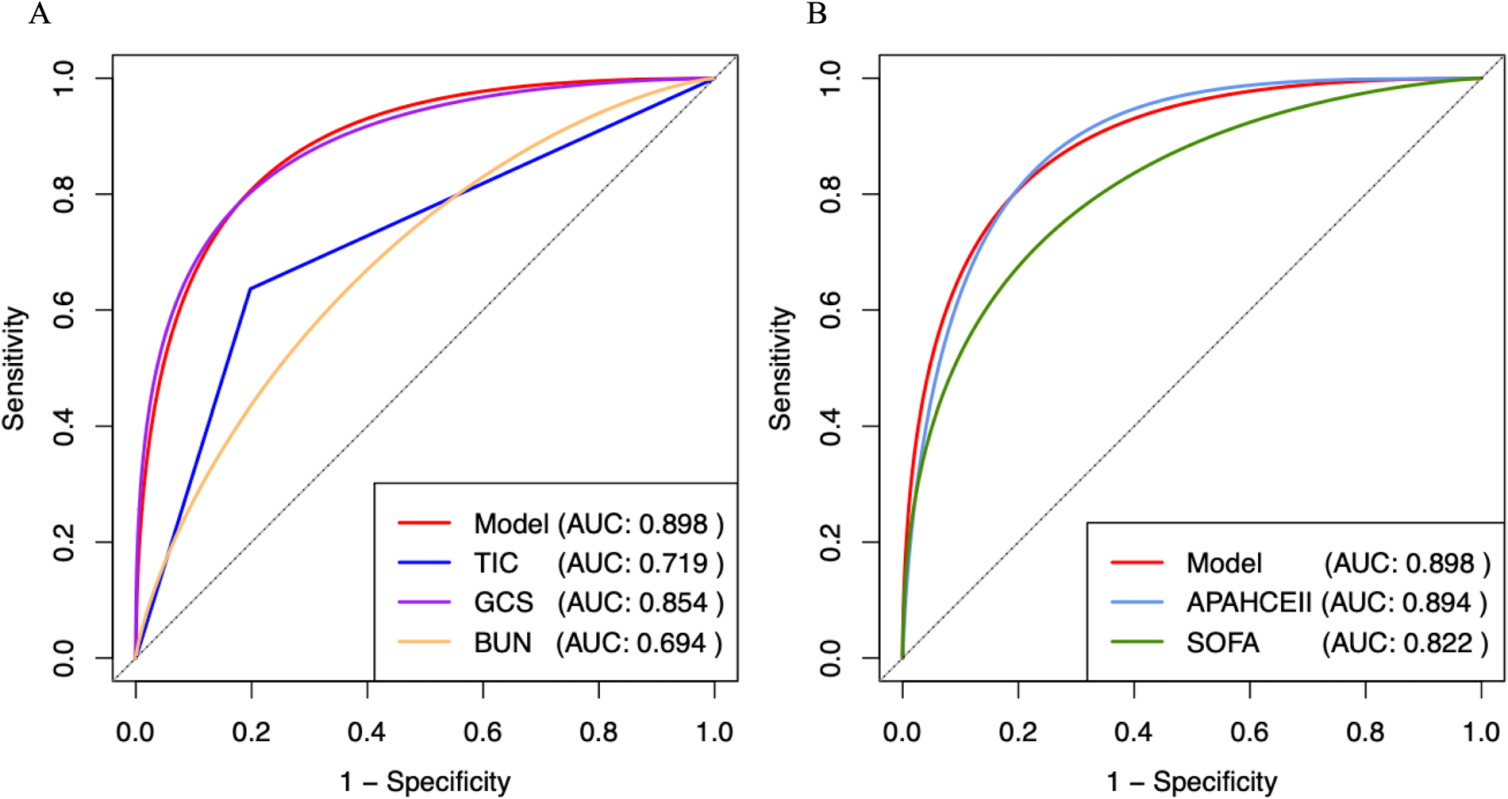
ROC curves of the prediction model and other factors. **A** ROC curve comparison of the prediction model with TIC, GCS, and BUN. **B** ROC curve comparison of the prediction model with APACHE II and SOFA scores

ROC curve analysis was conducted for the prediction model and each predictive factor to assess their performance in predicting hospital mortality (Fig. 3A). The AUC for TIC in predicting hospital mortality was 0.719 (95% CI 0.626–0.812), with a sensitivity of 63.64%, specificity of 80.22%, and a Youden index of 0.4386. The AUC for GCS score at admission was 0.854 (95% CI 0.782–0.926), with a sensitivity of 78.79%, specificity of 83.52%, and a Youden index of 0.6230. The AUC for BUN at admission was 0.694 (95% CI 0.590–0.798), with a sensitivity of 84.85%, specificity of 48.35%, and a Youden index of 0.3320. The combined regression model incorporating TIC, GCS, and BUN for predicting hospital mortality yielded an AUC of 0.898 (95% CI 0.834–0.962), with a sensitivity of 90.91%, specificity of 80.22%, and a Youden index of 0.7113. The prediction model demonstrated a significantly higher AUC compared to TIC and BUN (P < 0.001), but no statistical difference in AUC was observed between the prediction model and GCS score (P = 0.152) (Table 2).

**Table 2:** ROC curve comparison of the prediction model with TIC, GCS, and BUN

	AUC	95% CI	cutoff	Sensitivity (%)	Specificity (%)	Youden index
Prediction model	0.898^a,b,c^	0.834–0.962	0.28	90.91	80.22	0.7113
TIC	0.719^a^	0.626–0.812	1	63.64	80.22	0.4386
GCS	0.854^b^	0.782–0.926	5	78.79	83.52	0.6230
BUN	0.694^c^	0.590–0.798	4.7	84.85	48.35	0.3320

*TIC* trauma induced coagulopathy, *GCS* Glasgow Coma Scale, *BUN* blood urea nitrogen, *OR* odds ratio, *CI* confidence interval

^a^Difference detected by the DeLong's test for the ROC of the prediction model and TIC (P-value < 0.001)

^b^No difference detected by the DeLong's test for the ROC of the prediction model and GCS score (P-value = 0.152)

^c^Difference detected by the DeLong's test for the ROC of the prediction model and BUN (P-value < 0.001)

Additionally, ROC curve analysis was performed for APACHE II and SOFA scores to predict hospital mortality (Fig. 3B). The AUC for the SOFA score was 0.822 (95% CI 0.734–0.910), with a sensitivity of 75.76%, specificity of 81.32%, and a Youden index of 0.5708. The AUC for APACHE II score was 0.894 (95% CI 0.827–0.960) with a sensitivity of 81.82%, specificity of 86.81%, and a Youden index of 0.6863. When compared to traditional scoring systems, the AUC of the prediction model was not significantly different from that of the APACHE II score (P = 0.870), but it was higher than that of SOFA score (P = 0.037) (Table 3).

**Table 3:** ROC curve comparison of prediction model with APACHE II and SOFA scores

	AUC	95% CI	cutoff	Sensitivity (%)	Specificity (%)	Youden index
Prediction model	0.898^a,b^	0.834–0.962	0.28	90.91	80.22	0.7113
SOFA	0.822^a^	0.734–0.910	9	75.76	81.32	0.5708
APACHE II	0.894^b^	0.827–0.960	28	81.82	86.81	0.6863

*APACHE II* Acute Physiology and Chronic Health Evaluation II, *SOFA* Sequential Organ Failure Assessment, *AUC* area under the curve, *CI* confidence interval

^a^Difference detected by the DeLong's test for the ROC of the prediction model and SOFA score (P-value = 0.037)

^b^No difference detected by the DeLong's test for the ROC of the prediction model and APACHE II score (P-value = 0.870)

## Discussion

Multi-trauma, usually caused by high-energy impacts, represents some of the most severe injuries encountered in clinical practice, often involving multiple body loci and organs [14]. The mortality rate for such injuries is alarmingly high, frequently occurring within the first 1–6 h post-injury [9]. This urgency has led to the introduction of the “golden first hour” concept, emphasizing the critical need for prompt identification and treatment of high-risk patients within the first hour after injury to reduce clinical mortality and complication rates, thereby improving prognosis.

Given the complexity and high mortality of multi-trauma, accurate assessment upon emergency admission is essential. This study divided patients into survivor and non-survivor groups, examining clinical indicators to provide reference for early prediction, identification, and intervention in multi-trauma patients. The key findings are as follows: (1) TIC was more prevalent among non-survivors; (2) TIC and BUN were identified as independent risk predictors of hospital mortality, while GCS was identified as an independent protective predictor; (3) a nomogram incorporating TIC, BUN, and GCS was developed, demonstrating strong predictive performance and clinical impact.

TIC has a multifactorial pathogenesis, driven by tissue injury, bleeding, hypoperfusion, endothelial injury, and systemic inflammatory response [16]. Post-injury endothelial damage can lead to significant consumption of coagulation factors and activation of the fibrinolysis system [17]. Volume resuscitation may further dilute coagulation substances, while metabolic acidosis and hypothermia can diminish the activity of coagulation factors [18]. A previous study found that traumatic patients with TIC had higher blood transfusion requirements, more frequent hypotensive, tachycardic, and low oxygen saturation, and higher mortality [19]. Consistent with these findings, our study revealed that TIC occurred more frequently among non-survivors compared to survivors, highlighting TIC as a significant predictor of hospital mortality. The OR for TIC in this study was 4.238 (95% CI 1.46–12.28), and the AUC for predicting hospital mortality in patients with multi-trauma was 0.719 (95% CI 0.626–0.812), indicating a substantial increase in mortality risk associated with TIC.

The GCS score is a well-established measure of traumatic brain injury severity in trauma patients [20]. This study found that GCS score was significantly lower among non-survivors, ranging from 3 to 4 with a median of 3, compared to a median of 12 in survivors. Multivariate logistic analysis confirmed that GCS score was a reliable predictor of early mortality in trauma patients (OR 0.720, 95% CI 0.62–0.85), consistent with previous studies [21]. A lower GCS score indicates more severe traumatic brain injury (TBI), which correlates with both the severity of trauma-induced coagulopathy (TIC) and overall prognosis [22]. TBI can induce a hypocoagulable state partly due to the cerebral tissue releasing high concentrations of potent procoagulant molecules, such as phospholipids and brain-derived cellular microvesicles, which stimulate clotting that depletes clotting factors and provokes platelet inhibition [23]. Moreover, TBI also triggers endothelial injury, as evidenced by the release of von Willebrand factor, which further exacerbating the extent of TIC [24].

BUN was also identified as another independent risk factor for hospital mortality, with an OR of 1.397 (95% CI 1.09–1.78). As we know, BUN is a kidney injury index, reflecting the extent of organ ischemia and hypoxia, which is involved in the pathogenesis of TIC [25]. Furthermore, recent studies suggest that BUN levels may also be related to the inflammatory state of the body, which intricately interacts with the coagulation system [26, 27]. Taken together, elevated BUN levels may indicate kidney hypoperfusion and inflammation, underscoring the importance of monitoring BUN as an indicator of prognosis in muti-trauma patients.

The SOFA and APACHE II scores are widely used to evaluate organ dysfunction and mortality risk in trauma patients [28]. Similarly, our study found that both scores were effective in predicting hospital mortality, with AUCs of 0.822 (95% CI 0.734–0.910) and 0.894 (95% CI 0.827–0.960), respectively. While the SOFA and APACHE II scores provide comprehensive assessments of organ function and disease severity, the prediction model developed in this study offers a simpler and more practical tool for rapid bedside evaluation. By focusing on easily obtainable parameters such as TIC, BUN, and GCS score, this prediction model can quickly assess the condition and prognosis of multi-trauma patients upon admission. Although it may not be as detailed as the SOFA or APACHE II scores, its simplicity enhances its reproducibility and suitability for daily monitoring, placing less burden on patients. Moreover, both GCS and BUN are closely associated with the development of TIC, highlighting the rationale for combining these factors in the prognosis evaluation of multi-trauma patients.

Despite the promising findings, this study has several limitations, primarily due to its single-center, retrospective design with a limited sample size. Future research should address these limitations by conducting multicenter, prospective studies to validate the performance of the predictive model and ensure its broader applicability.

## Conclusions

Our study identified TIC, BUN, and GCS score as independent predictors of hospital mortality in multi-trauma patients. Based on these predictors, we established a nomogram for prognostic prediction, aimed at early identification and prompt treatment of patients with potentially poor prognoses. However, future studies are needed to validate the accuracy and reliability of this prediction model in diverse clinical settings and larger, multicenter populations.

## Supplementary Information

Below is the link to the electronic supplementary material.

Supplementary file1 (DOCX 46 KB)

## Data Availability

The datasets generated during and/or analyzed during the current study are available from the corresponding author on reasonable request.
