# Supplementary material for: A nomogram based on trauma-induced coagulopathy for predicting hospital mortality in multi-trauma patients: a retrospective study
Source: Intern Emerg Med. 2025 Jan 28;20(8):2513–21. doi: 10.1007/s11739-025-03867-w (PMC12672700; doi:10.1007/s11739-025-03867-w)
Supplement: Supplementary file 1 — Supplementary file1 (DOCX 46 KB) [file 11739_2025_3867_MOESM1_ESM.docx]

**Title:** A nomogram based on Trauma-induced coagulopathy for predicting hospital mortality in multi-trauma patients: a retrospective study

**Journal:** Internal and Emergency Medicine

**Author and affiliation:** Shaochuan Chen^1†^, Jiale Yang^2,3†^, Xuezhi Shi^3^, Anwei Liu^3^, Guodong Lin^3^, Huasheng Tong^3^*

^1^Department of emergency medicine, Huiyang Sanhe Hospital, Huizhou, China

^2^Guangzhou University of Chinese Medicine, Guangzhou, China.

^3^Department of Intensive Care Unit, General Hospital of Southern Theatre Command of PLA, Guangzhou, China.

^†^These authors contributed equally to this work and should be considered co-first authors

*** Correspondence:**
Huasheng Tong
[fimmuths@163.com](mailto:fimmuths@163.com)

**Table S1** Univariate logistic regression analyses of factors associated with hospital mortality in patients with multi-trauma

| Variables | OR | 95%CI | P-value |
| --- | --- | --- | --- |
| Age | 1.05 | 1.02-1.08 | 0.002 |
| Temperature |  |  |  |
| ≤35℃ | 20.50 | 5.41-77.67 | <0.001 |
| 35.1-36.2℃ | 2.41 | 0.56-10.37 | 0.237 |
| ≥36.3℃ | ref | ref |  |
| MAP（mmHg） | 0.97 | 0.95-0.99 | 0.001 |
| Lac (mmol/L) | 1.16 | 1.04-1.30 | 0.006 |
| PLT（×10^9^/L） | 0.99 | 0.99-1.00 | 0.003 |
| APTT（s） | 1.05 | 1.02-1.08 | 0.003 |
| PT（s） | 1.32 | 1.15-1.50 | <0.001 |
| INR | 17.40 | 4.09-73.92 | <0.001 |
| Fib（g/L） | 0.37 | 0.20-0.69 | 0.002 |
| Scr（μmol/L） | 1.01 | 1.00-1.02 | 0.013 |
| BUN（mmol/L） | 1.32 | 1.10-1.58 | 0.003 |
| TIC（%） | 7.10 | 2.95-17.06 | <0.001 |
| Mechanical ventilation（%） | 18.90 | 4.27-83.70 | <0.001 |
| Vasoactive drugs（%） | 10.84 | 3.81-30.84 | <0.001 |
| CRRT（%） | 12.43 | 3.64-42.43 | <0.001 |
| Platelet transfusion（%） | 5.19 | 1.86-14.46 | 0.002 |
| ISS score | 1.08 | 1.04-1.12 | <0.001 |
| GCS score | 0.69 | 0.58-0.81 | <0.001 |
| APACHE II score | 1.22 | 1.14-1.31 | <0.001 |
| SOFA score | 1.37 | 1.20-1.55 | <0.001 |
| Degree of Coma |  |  |  |
| Light（%） | ref | ref |  |
| Moderate（%） | 7.17 | 0.6-85.95 | 0.120 |
| Severe（%） | 35.83 | 4.65-275.85 | <0.001 |
| Degree of injury |  |  |  |
| Moderate（%） | ref | ref |  |
| Severe（%） | 10.20 | 1.32-79.03 | 0.026 |

MAP: mean artery pressure; Lac: lactate; PLT: platelet; APTT: activated partial thromboplastin time; PT: prothrombin time; INR: international normalized ratio; Fib: fibrinogen; Scr: serum creatinine; BUN: blood urea nitrogen; TIC: trauma induced coagulopathy; CRRT: Continuous Renal Replacement Therapy; ISS: Injury severity score; GCS: Glasgow Coma Scale; APACHE II: Acute Physiology and Chronic Health Evaluation II; SOFA: Sequential Organ Failure Assessment; Degree of Coma: Light, GCS 13-15; Moderate, GSC 9-12; Severe, GCS ≤ 8; Degree of injury: Moderate, ISS 16-24; Severe, ISS ≥ 25. OR: odds ratio; CI: confidence interval.

**Table S2** The prediction model based on TIC, GCS, and BUN

| Variables | β | OR | 95%CI | P-value |
| --- | --- | --- | --- | --- |
| TIC | 1.444 | 4.238 | 1.46-12.28 | 0.008 |
| GCS | -0.329 | 0.720 | 0.61-0.85 | <0.001 |
| BUN | 0.334 | 1.397 | 1.09-1.78 | 0.007 |
| Constant | -1.444 | 0.236 | 0.04-1.36 | 0.105 |

TIC: trauma induced coagulopathy; GCS: Glasgow Coma Scale; BUN: blood urea nitrogen. OR: odds ratio; CI: confidence interval.
